# Creating an Improved Diatoxanthin Production Line by Knocking Out *CpSRP54* in the *zep3* Background in the Marine Diatom *Phaeodactylum tricornutum*

**DOI:** 10.3390/md23110419

**Published:** 2025-10-29

**Authors:** Charlotte Volpe, Zdenka Bartosova, Ralph Kissen, Per Winge, Marianne Nymark

**Affiliations:** 1 Department of Fisheries and New Biomarine Industry, SINTEF Ocean, Trondheim 7010, Norway; charlotte.volpe@sintef.no; 2 Department of Biology, Norwegian University of Science and Technology, Trondheim 7491, Norway; zdenka.bartosova@ntnu.no (Z.B.); ralph.kissen@ntnu.no (R.K.); per.winge@ntnu.no (P.W.)

**Keywords:** diatoms, *Phaeodactylum tricornutum*, high-value products, diatoxanthin, CRISPR/Cas9 gene editing

## Abstract

Diatoxanthin is a photoprotective carotenoid found in a few groups of microalgae displaying in vitro anti-inflammatory and anti-cancer properties, making it a promising candidate for nutraceutical, pharmaceutical, and cosmetic applications. However, large-scale production is currently nonexistent because of two major challenges: Instability during microalgae harvesting, where diatoxanthin is rapidly converted back to its inactive precursor diadinoxanthin under non-stressful light conditions, and dependence on prolonged exposure to high-intensity light, which is costly and technically challenging during indoor high-cell-density cultivation. The first limitation was previously addressed by knocking out zeaxanthin epoxidase 3 (ZEP3) in the marine diatom *Phaeodactylum tricornutum*, resulting in a mutant that stabilized diatoxanthin under non-stressful light conditions. Here, we report an improved diatoxanthin production line where both of the described challenges have been overcome. This was achieved by creating *P. tricornutum* mutants where the phenotype of the *zep3* mutant was combined with the light-sensitive phenotype of the *chloroplast signal recognition particle 54* (*cpsrp54*) mutant. Growth rates were maintained at wild-type levels at light intensities ≤ 150 µmol photons m^−2^ s^−1^ in the *zep3cpsrp54* mutants, but prolonged medium light exposure resulted in a 1.5- and 7-fold increase in diatoxanthin concentration compared with *zep3* and wild-type, respectively. When returned to low light, the *zep3cpsrp54* cultures retained ~80% of their accumulated diatoxanthin. The improved production lines allow for diatoxanthin accumulation without the use of high-intensity light and with limited loss of diatoxanthin when returned to non-stressful light conditions.

## 1. Introduction

Carotenoids represent the most widespread group of naturally occurring pigments in nature; they are synthesized by all photosynthetic organisms and have essential roles in photosynthesis and photoprotection [1,2]. Carotenoids are a group of fat-soluble compounds with unique spectral properties, causing them to exhibit a wide array of colors from yellow to red [1,3]. The basic carotenoid structure consists of eight isoprenoid units with a series of conjugated double bonds, and they are divided into hydrocarbons (carotenes) and their oxygenated derivatives (oxycarotenoids or xanthophylls) [1,4]. Because of the carotenoids’ potential for health-promoting biological actions in humans, primarily through their strong antioxidant activity, carotenoid research has received much attention in recent decades [5,6,7,8]. Several studies have reported positive effects of carotenoids as preventives against diseases such as cancer [9], diabetes [10], neurodegenerative [11], and cardiovascular disorders [12], increasing the interest in industrial-scale production of these compounds [13,14,15]. Currently, only a subset of carotenoids, which include β-carotene, zeaxanthin, astaxanthin, and fucoxanthin (Fx), among others, are produced industrially, mainly for their use as dietary supplements, as food and feed colorants, and in cosmetics and pharmaceuticals. To date, the global carotenoid market size ranges from USD 1.5 to 2.5 billion, depending on the source (Grand View Research, MarketsandMarkets, Research and Markets). The increasing commercial applications of these bioactive compounds have led to a growing demand, also for achieving industrial production of other carotenoids with high potential. Currently, the bulk of industry-produced carotenoids are synthesized chemically [16], though a small portion of carotenoids is obtained naturally from plants or algae [17,18,19]. However, studies have shown that naturally produced pigments might be safer, more bioavailable, and exhibit a higher biological activity compared with their synthetic counterpart [20,21,22,23], indicating the need to shift to naturally produced pigments, especially if the product is marketed as a functional ingredient. Since micro- and macroalgae represent a primary source of natural carotenoids, carotenoid extraction from cultivated algae may help in overcoming problems with balancing the supply of and demand for these products whilst maintaining their functional properties. 

Recent studies have reported that diatoxanthin (Dtx), a carotenoid found in diatoms and a few other groups of microalgae [24], possesses antioxidant, anti-inflammatory, and anti-cancer properties [25,26,27,28,29]. Research indicates that Dtx has the potential to counteract the development of cancer cells in vitro by inducing oxidative stress-mediated cell death, highlighting the potential for its use as a therapeutic agent in the future [27,28]. Dtx is a photoprotective carotenoid of low abundance that, together with diadinoxanthin (Ddx), comprises the xanthophyll cycle [24]. Upon sudden high-light exposure, Ddx is rapidly converted to Dtx by violaxanthin de-epoxidase (VDE). The conversion from Ddx to Dtx, together with a low pH in the thylakoid lumen and the presence of specific light-harvesting complex (LHC) proteins (the LHCX proteins in diatoms), causes activation of the photoprotective mechanism non-photochemical quenching (NPQ), enabling safe dissipation of excessively absorbed light energy as heat [24]. The back conversion to Ddx is triggered as soon as the algae are removed from high light and is catalyzed by zeaxanthin epoxidase (ZEP) [24]. 

The therapeutic bioactivities indicated by recent in vitro research suggest that Dtx might become a compelling candidate to be used in nutraceutical, pharmaceutical, and cosmetic applications. Currently, Dtx is only commercially available in very low concentrations and quanta as pigment standards. The rapid loss of Dtx in low light has been identified as one of the major challenges for industrial-scale production of this carotenoid since Dtx, produced as a response to high light treatment, will rapidly be converted to Ddx during harvesting of the algae biomass in non-stressful light conditions [30]. Large-scale harvesting of microalgae is a time-consuming process taking place in low light or darkness [31]. To solve the above-described challenge, the enzyme responsible for the de-epoxidation of Dtx to Ddx, the zeaxanthin epoxidase 3 (ZEP3), was recently identified in the diatom *P. tricornutum* [30,32]. Loss of functional ZEP3 enzymes resulting from the creation of CRISPR-Cas9 induced knockout (KO) mutations in the *ZEP3* gene, stabilized high-light accumulated Dtx for up to 2 h after the *P. tricornutum* cultures had been removed from the high-light source [30], suggesting that *P. tricornutum zep3* KO mutants could function as a commercial production line of Dtx. Another challenge connected to large-scale microalgae production of Dtx is the dependency on prolonged exposure to high-intensity light to achieve commercially interesting levels of Dtx [30,33]. This can be technically challenging as high cell density cultures are characterized by limited light penetration due to self-shading and can be costly in countries where algae production is dependent on artificial light [34,35].

*P. tricornutum* is among the top-produced microalgae species in Europe [36], it is of interest as a producer of Fx [37,38], and it has a high potential as a feed ingredient within aquaculture because of its high digestibility and nutritional profile [39,40]. The high potential of this diatom to be a source of valuable microalgae products, in combination with a fully sequenced genome and an advanced molecular toolbox [41,42,43,44,45], makes it an obvious choice for further strain optimization to fulfil its industrial potential. In this context, we aimed to make an improved Dtx production line no longer dependent on high-intensity light to trigger production of the pigment. Loss of the chloroplast signal recognition particle 54 (CpSRP54) protein in *P. tricornutum* has previously been reported to cause light sensitivity and increased production of Dtx at moderate light conditions [46]. Diatom CpSRP54 proteins play a role in the CpSRP pathway, targeting proteins to thylakoid membranes, but in contrast to plants and green algae, diatom CpSRP54 is not involved in the insertion of LHC proteins [46,47,48]. Previous work showed that *P. tricornutum cpsrp54* KO mutants display WT levels of Fx and Chls and normal growth rates in non-stressful light conditions [46]. Based on the reported phenotype of the *zep3* and *cpsrp54* single KO mutants, we hypothesized that creation of *zep3cpsrp54* double KO mutants could result in an improved Dtx production strain that is independent of high light to trigger Dtx production and where accumulated Dtx is stable during harvesting of the algae biomass.

## 2. Results and Discussion

### 2.1. Generation of zep3cpsrp54 Double KO Mutants by CRISPR/Cas9 Gene Editing of the CpSRP54 Gene in zep3 Mutants

**Figure 1:**
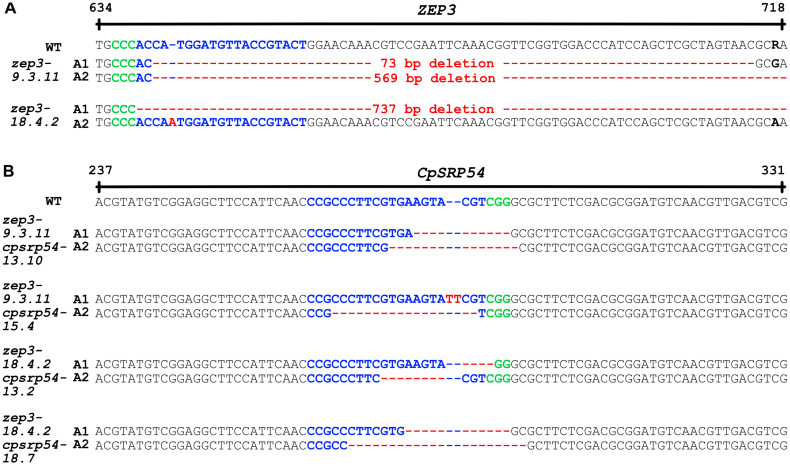
Overview of indels in the *zeaxanthin epoxidase 3* (*ZEP3)* and *chloroplast signal recognition particle 54* (*CpSRP54)* genes. (**A**) Indels in the *ZEP3* gene in the *zep3* single knockout (KO) lines that were subjected to CRISPR/Cas9-mediated mutagenesis to create *zep3cpsrp54* double KO lines. (**B**) Indels in the *CpSRP54* gene in the *zep3csrp54* double KO lines. Blue characters: target sequences; red characters: indels; green characters: protospacer adjacent motifs (PAMs). The PAM for the *ZEP3* target site is located on the reverse strand.

*Zep3cpsrp54* double KO mutants were generated by introducing mutations in the *CpSRP54* gene in cells derived from previously published *zep3* mutant lines using the CRISPR/Cas9 technology [30,44]. The use of bacterial conjugation as a delivery method of the CRISPR/Cas9 components can prevent incorporation of foreign DNA into the *P. tricornutum* genome [44]. The two previously published *zep3* lines were assumed to contain bi-allelic mutations in the *ZEP3* gene, but this was not verified by Græsholt et al. [30]. Only one mutated allele in each of the two *zep3* lines was identified by genomic PCR and Sanger sequencing, but bi-allelic mutations were assumed since non-mutated *ZEP3* gene sequences were absent. After isolating *zep3* cells no longer able to grow on selective media, indicating a loss of the CRISPR/Cas9 plasmid (Supplementary Figure S1) [44]. Nanopore genome sequencing of two representative lines (*zep3-9.3.11* and *zep3-18.4*; derived from *zep3-1* and *zep3-2* in Græsholt et al. [30], respectively) confirmed loss of the plasmid and verified that the *zep3* lines contained bi-allelic mutations in the target gene. The 73 bp deletion in allele 1 (A1) in the *zep3-9.3.11* line was verified [30], and a larger deletion of 569 bp in allele 2 (A2) was revealed (Figure 1A). Similarly, the previously reported 1 bp insertion in A2 in *zep3-18.4.2* was verified [30], and a deletion of 737 bp was identified in A1 through genome sequencing (Figure 1A). Subsequent CRISPR/Cas9 editing successfully introduced small bi-allelic mutations in *CpSRP54* in both *zep3-9.3.11* and *zep3-18.4.2* backgrounds [44]. Four *zep3cpsrp54* mutant lines were selected for phenotypic characterization. An overview of the indels present in the *CpSRP54* gene in the selected double KO mutants is presented in Figure 1B. 

### 2.2. Physiological Features of the zep3cpsrp54 Double KO Mutants

**Figure 2:**
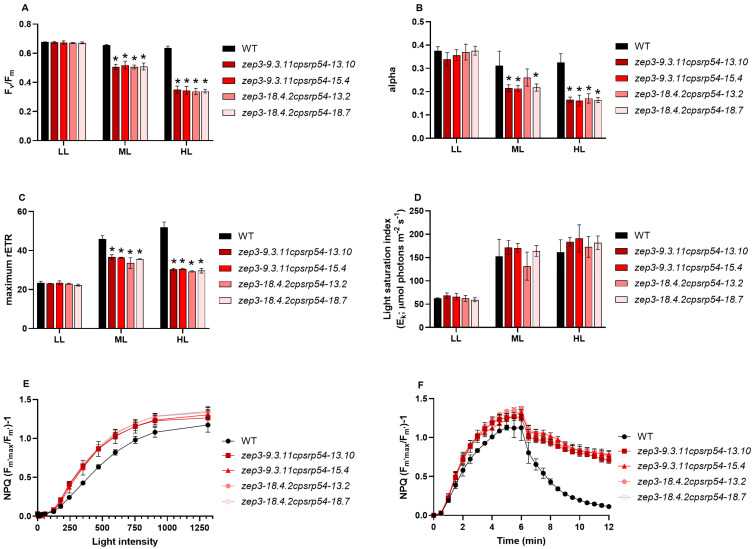
Photosynthetic performance of *zep3cpsrp54* double KO mutants compared with wild-type (WT). (**A**) The photosynthetic (PSII) efficiency (F_v_*/*F_m_), (**B**) the maximum light utilisation coefficient (alpha), (**C**) the photosynthetic capacity (maximum relative electron transport rate (rETR_max_)), (**D**) the light saturation index (*E*_k_) in WT and four *zep3cpsrp54* double KO mutants acclimated to either low light (LL; 35 µmol photons m^−2^ s^−1^), medium light (ML; 200 µmol photons m^−2^ s^−1^) or high light (HL; 450 µmol photons m^−2^ s^−1^). Asterisks describe significant differences between mutants and WT as indicated by two-way ANOVA with Dunnett’s multiple comparison tests (*p* < 0.05). Non-photochemical quenching (NPQ) was calculated both as (**E**) a function of increasing blue light intensity (0-1313 µmol photons m^−2^ s^−1^) and as (**F**) a function of time where the cells were exposed to 6 min of high intensity blue light (470 µmol photons m^−2^ s^−1^), immediately followed by a 6 min recovery period in low intensity blue light (8 µmol photons m^−2^ s^−1^). NPQ measurements were performed on LL-acclimated cells after a 5 min dark incubation period. All results are presented as the mean of three biological replicates  ±  SD.

Both *P. tricornutum zep3* and *cpsrp54* single KO mutants have altered photophysiological features [30,32,43,46,49]. The *zep3* mutants are unable to relax the NPQ response after induction [30,32,49], whereas the *cpsrp54* mutants display elevated NPQ levels and reduced photosynthetic performance at increased light intensities compared with WT [43,46]. To determine whether the *zep3cpsrp54* double KO combined these characteristics, the photophysiological responses to low light (LL; 35 μmol photons m^−2^ s^−1^), medium light (ML; 200 μmol photons m^−2^ s^−1^), and high light (HL; 450 μmol photons m^−2^ s^−1^) were compared with WT responses. Under LL, the double KO exhibited photosynthetic performance comparable to WT and the two single mutants (Figure 2A–D) [30,46], indicating that the double KO does not impair photosynthesis under these conditions. In contrast, the double KO mutants treated with ML and HL displayed decreased photosynthetic (PSII) efficiency (F_v_/F_m_), maximum light utilisation coefficient (alpha), and photosynthetic capacity (maximum relative electron transport rate (rETRmax)), and elevated NPQ levels compared with WT (Figure 2A–C,E,F) consistent with the phenotype previously described for *cpsrp54* mutants [46]. As for the *zep3* single KO mutants [30], NPQ levels stayed high during the relaxation phase in very low intensity light (8 µmol photons m^−2^ s^−1^ of blue light) in the double KO mutants (Figure 2F). NPQ is known to be linearly correlated with the Dtx concentration in pennate diatoms [50,51,52], meaning that elevated NPQ levels observed during the relaxation phase for the double KOs imply that Dtx is not immediately converted back to Ddx when the cells are no longer experiencing high light stress. Consistent with results previously reported for *zep3* single mutants [30,49], the double KOs displayed an abrupt decline in NPQ during the first 30 seconds of recovery, followed by relatively stable high NPQ levels at the subsequent measuring points. This decrease is assumed to be independent of Dtx, but the mechanism behind the phenomenon is unknown [49].

**Figure 3:**
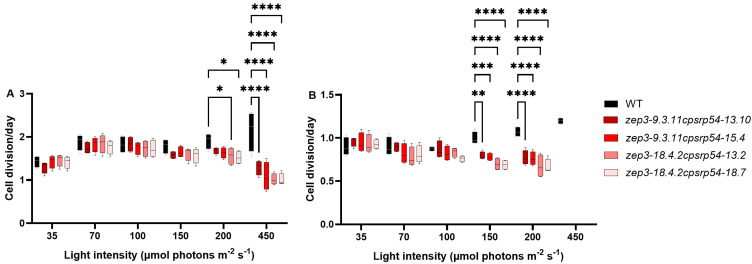
Growth rates of *zep3cpsrp54* double KO mutants compared with WT at different light intensities and temperatures. WT and four *zep3cpsrp54* mutant lines were cultivated at 35, 70, 100, 150, 200, and 450 μmol photons m^−2^ s^−1^ of white light at (**A**) 15 °C and (**B**) 10 °C. The maximum number of cell divisions per day during the exponential phase was calculated from four biological replicates from each line. Asterisks describe significant differences between *zep3cpsrp54* and WT as indicated by two-way ANOVA with Dunnett’s multiple comparison tests (*p *< 0.05).

The growth rate of *zep3* mutants has been reported to be lower than WT in a variable light regime, but not in continuous light of either LL or HL [30,32]. In contrast, the *cpsrp54* mutants display a reduced cell division rate at increased light intensities [46]. To assess the effect of increasing light intensities on the growth rate of the *zep3cpsrp54* mutants, the double KOs were cultivated under a range of continuous white light intensities at 10 °C and 15 °C, temperatures relevant for large-scale cultivation in Nordic conditions [34]. Growth rates were also negatively affected by increasing light intensities in the double KO mutants, but significant differences between the double KO mutant lines and WT were only found at light intensities of 200 μmol photons m^−2^ s^−1^ or higher at 15 °C (Figure 3A). Growth rates were overall slower at 10 °C compared with 15 °C (Figure 3), likely because of a general slowdown in enzymatic processes, including enzymes in the Calvin cycle [53,54,55]. The lower temperature also enhanced the light sensitivity of the double KOs (Figure 3B). Significantly reduced cell division rates in the double KOs compared with WT were evident already at 150 μmol photons m^−2^ s^−1^ or higher at 10 °C, and no cell divisions occurred in the double KOs at 450 μmol photons m^−2^ s^−1^ when cultivated at this temperature (Figure 3B). However, the double KO mutants managed a few cell divisions during the 1-week acclimation period at 450 μmol photons m^−2^ s^−1^ at this temperature before the start of the growth experiments. The explanation for the lack of cell division during the growth experiment is likely connected to the low cell concentration (30,000 cells/mL) used as a starting concentration. In a low-density culture, the light exposure for each cell will be high and increase the chances for photodamage to occur when the light intensity is high. The light sensitivity in *P. tricornutum* cells lacking the CpSRP54 protein is suggested to be caused by an inefficient replacement of damaged PSII core proteins, causing a slower rate of PSII repair [46]. This effect might be strengthened with a decrease in temperature since PSII repair is known to slow down at low temperatures [56]. In contrast with the double KO mutants, the growth rate of the WT cultures showed the highest cell division rates at the highest light intensities at both temperatures. Moreover, HL-acclimated WT cultures also maintained F_v_*/*F_m_ values comparable to those measured in LL (Figure 2A), indicating that the rate of photodamage did not exceed the rate of PSII repair in WT cells.

### 2.3. Induction of NPQ in zep3cpsrp54 Compared with Single Mutants and WT Using Different Light Intensities

**Figure 4:**
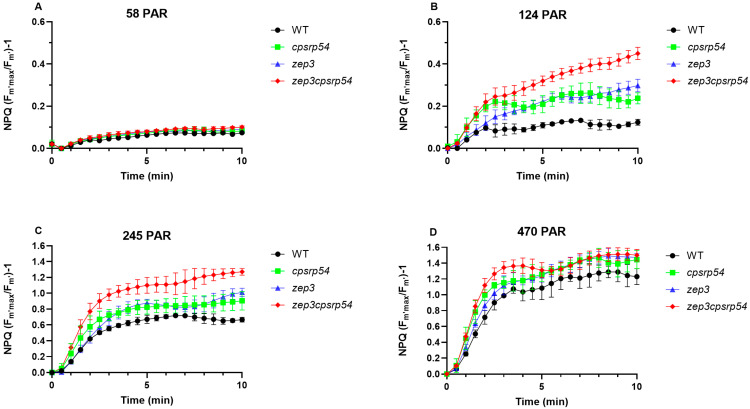
NPQ induction in *cpsrp54*, *zep3*, *zep3cpsrp54*, and WT as a response to constant light of different light intensities. NPQ was calculated as a function of time where the cells were exposed to (**A)** 58 µmol photons m^−2^ s^−1^, (**B**) 124 µmol photons m^−2^ s^−1^, (**C**) 245 µmol photons m^−2^ s^−1^, and (**D**) 470 µmol photons m^−2^ s^−1^ of blue light for 10 min. Two different lines were included in the experiment for *cpsrp54* (*cpsrp54-11*, *cpsrp54-20*) and *zep3* (*zep3-9.3.11*, *zep3-18.4.2*), and four different lines were included for *zep3cpsrp54* (*zep3-9.3.11cpsrp54-13.10*, *zep3-9.3.11cpsrp54-15.4*, *zep3-18.4.2cpsrp54-13.2*, *zep3-18.4.2cpsrp54-18.7*). Three biological replicates were included for each of the mutant lines, meaning that the total number of samples was *n* = 6 for *zep3* and *cpsrp54*; *n* = 12 for *zep3cpsrp54*; and *n* = 3 for WT. The graphs are based on the means of all lines and replicates for each mutant or WT. Data for the individual lines can be found in Supplementary Figure S2. Significant differences between *zep3cpsrp54* and the other lines for each time point and light intensity were investigated by performing a two-way ANOVA with Dunnett’s multiple comparison tests. The results of the statistical analyses are included in Supplementary Table S1.

The initial investigation of the photophysiological performance and growth at different light intensities suggested that the creation of an HL-sensitive mutant that retains accumulated Dtx had been successful, but further characterization of the phenotype was needed to determine if the *zep3cpsrp54* double KOs could outperform the *zep3* single KO mutants as a production line of Dtx. The photophysiological characterization of the double KOs revealed that NPQ was more strongly induced in those lines compared with WT, already at intensities around 200 μmol photons m^−2^ s^−1^ of blue light (Figure 2E). To further explore the potential of the *zep3cpsrp54* double KOs to produce Dtx, NPQ induction was compared among double KO mutants, the two single mutants (*zep3* and *cpsrp54*), and WT under constant blue light of different intensities (Figure 4; notice the different maximum values on the *y*-axis in Figure 4A,B compared with Figure 4C,D). Across all treatments WT displayed the weakest NPQ induction, the *zep3* and *cpsrp54* single mutants showed an intermediate response, whereas the double KOs displayed significantly higher NPQ values after 10 min of light exposure for all light intensities except for the highest one (Figure 4; Supplementary Table S1). 10 min exposure to 470 μmol photons m^−2^ s^−1^ of blue light triggered both mutants and WT to reach their maximum capacity for NPQ (Figure 4D). The largest differences in NPQ development between double KOs and the other lines were found as a response to exposing the algae to 124 μmol photons m^−2^ s^−1^ of blue light. This light intensity caused NPQ to reach 3.6 times higher values in double KOs compared with WT at the end of the light exposure period, and 1.5- and 1.9-times higher levels than in *zep3* and *cpsrp54*, respectively (Figure 4B). NPQ was rapidly activated during the first 2 min of light exposure in both the double KO and the *cpsrp54* single mutants, but whereas the NPQ levelled out after the initial rapid rise in the *cpsrp54* mutants, NPQ continued to rise during the entire light exposure period in the double KOs (Figure 4B). The slow rise in NPQ until the end of the experimental period (although to a lower level than in the double KOs) was also observed in the *zep3* mutant, showing again the combined features of both *cpsrp54* and *zep3* single KO mutants in the double KOs (Figure 4B). The generally stronger induction of NPQ in the double KOs compared with *zep3* lines at low to intermediate light intensity levels implies that high-intensity light might not be needed for efficient Dtx production in the double KO lines, and that using double KOs for production of Dtx might be advantageous over the use of *zep3* lines.

### 2.4. Dtx Production in zep3cpsrp54 Double KO Compared with Single Mutants and WT as a Result of Prolonged ML Exposure

**Figure 5:**
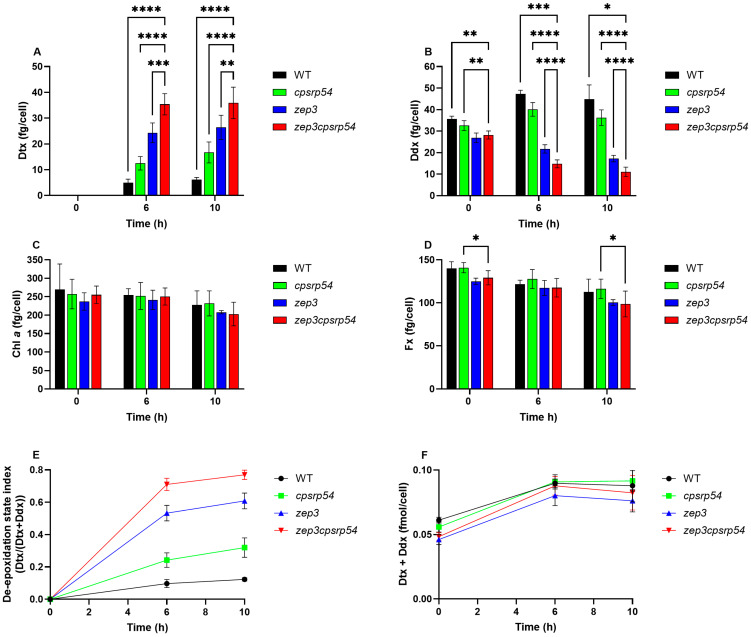
Pigment levels and de-epoxidation state (DES) index in LL-acclimated *cpsrp54*, *zep3*, *zep3cpsrp54,* and WT cells after prolonged ML treatments. Cellular pigment contents (fg/cell) are shown for (**A**) Diatoxanthin (Dtx), (**B**) Diadinoxanthin (Ddx), (**C**) Chlorophyll (Chl) *a*, and (**D**) Fucoxanthin (Fx) in *cpsrp54*, *zep3*, *zep3cpsrp54*, and WT after exposure of LL (35 µmol photons m^−2^s^−1^) acclimated cells (0 h) to 6 and 10 h of ML (200 µmol photons m^−2^s^−1^). Two independent mutant lines were included in the experiment for *cpsrp54* (*cpsrp54-11*, *cpsrp54-20*) and *zep3* (*zep3-9.3.11*, *zep3-18.4.2*), and four independent lines were included for *zep3cpsrp54* (*zep3-9.3.11cpsrp54-13.10*, *zep3-9.3.11cpsrp54-15.4*, *zep3-18.4.2cpsrp54-13.2*, *zep3-18.4.2cpsrp54-18.7*). Three biological replicates were included for each of the independent mutant lines, meaning that the total number of samples was *n* = 6 for *zep3* and *cpsrp54*; *n* = 12 for *zep3cpsrp54;* and *n* = 3 for WT. The graphs are based on the means of all lines and replicates for each mutant or WT. Asterisks for figures (**A**–**D**) describe significant differences between *zep3cpsrp54* and the other lines as indicated by two-way ANOVA with Dunnett’s multiple comparison tests (*p *< 0.05). (**E**) DES index (DES = Dtx/(Dtx + Ddx)) calculated from data shown in (**A**,**B**) after conversion to fmol/cell. (**F**) Changes in the total pool of Dtx + Ddx (fmol/cell) as a function of time after the shift from LL to ML. Data for the individual lines can be found in Supplementary Figure S3.

The results described in Section 2.3 indicate that NPQ, and therefore presumably also Dtx production, can be triggered to a relatively high level without the use of high light intensities in *zep3cpsrp54* double KOs. To test this hypothesis, we exposed the same mutant lines as used for the NPQ experiments to ML for 6 h and 10 h. The time points were based on when *P. tricornutum cpsrp54* mutants and/or WT showed the highest level of Dtx production and lowest level of F_v_*/*F_m_ when exposed to light stress in previous studies [33,46]. Consistent with NPQ data, pigment analysis revealed that ML was sufficient to induce substantial Dtx accumulation in the double KO, and that the double KOs contained the highest concentrations of Dtx, followed, in decreasing order, by *zep3*, *cpsrp54,* and WT (Figure 5A), confirming a cumulative effect of combining the two mutations. The opposite trend was observed for the Ddx concentration (Figure 5B). The Dtx production in the double KOs clearly outperformed the production in the single KO lines, demonstrating Dtx concentrations that were 46% and 36% higher than in *zep3* lines after 6 h and 10 h of ML treatment, respectively. The Dtx concentration in the double KOs after 6 h in ML reached similar levels as previously reported for *zep3* single mutants exposed to HL for 2 h [30]. Even though the concentration of Dtx differed significantly between mutant lines, they were highly similar for the same mutant line at both 6 h and 10 h (Figure 5A). These results suggest that exposing the cells to 10 h of ML to produce Dtx would be a waste of light energy if using the double KOs to produce Dtx in a future large-scale production setting. The changes in the de-epoxidation state (DES) index and the total pool of photoprotective pigments (Dtx + Ddx) were also minimal between the two ML light exposure time points (Figure 5E,F). As previously reported for the *cpsrp54* and the *zep3* single mutants, Chl *a* and Fx concentrations were highly similar between all lines and in all conditions (Figure 5C,D) [30,46]. 

### 2.5. Dtx Production in zep3cpsrp54 Double KO Compared with Single Mutants and WT as a Result of Short-Term ML Exposure

**Figure 6:**
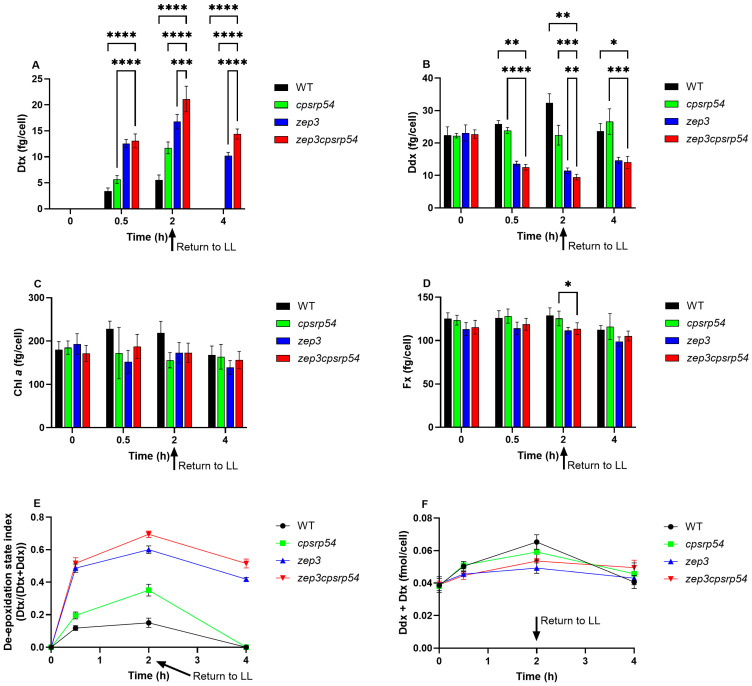
Pigment levels and DES index in LL-acclimated *cpsrp54*, *zep3*, *zep3cpsrp54*, and WT cells after a shift to ML followed by being returned to LL. Cellular pigment contents (fg/cell) are shown for (**A**) Dtx, (**B**) Ddx, (**C**) Chl *a*, and (**D**) Fx in *cpsrp54*, *zep3*, *zep3cpsrp54,* and WT after exposure of LL (35 µmol photons m^−2^ s^−1^) acclimated cells (0 h) to 0.5 and 2 h of ML (200 µmol photons m^−2^ s^−1^). The cultures were returned to LL for 2 h after 2 h of ML treatment (corresponding to the 4 h time point in the above figure). Two different lines were included in the experiment for *cpsrp54* (*cpsrp54-11*, *cpsrp54-20*) and *zep3* (*zep3-9.3.11*, *zep3-18.4.2*), and four different lines were included for *zep3cpsrp54* (*zep3-9.3.11cpsrp54-13.10*, *zep3-9.3.11cpsrp54-15.4*, *zep3-18.4.2cpsrp54-13.2*, *zep3-18.4.2cpsrp54-18.7*). Three biological replicates were included for each of the mutant lines, meaning that the total number of samples was *n* = 6 for *zep3* and *cpsrp54*; *n* = 12 for *zep3cpsrp54;* and *n* = 3 for WT. The graphs are based on the means of all lines and replicates for each mutant or WT. Asterisks for figures (**A**–**D**) describe significant differences between *zep3cpsrp54* and the other lines as indicated by two-way ANOVA with Dunnett’s multiple comparison tests (*p* < 0.05). (**E**) DES index (DES = Dtx/(Dtx + Ddx)) calculated from data shown in (**A**,**B**) after conversion to fmol/cell. (**F**) Changes in the total pool of Dtx + Ddx (fmol/cell) as a response to the different light treatments.

A second experiment was conducted to evaluate whether shorter exposure times (0.5 and 2 h) of ML could be sufficient to induce high levels of Dtx in the double KOs. The pigment analysis of LL-acclimated cultures exposed to ML for 0.5 and 2 h (Figure 6A–D) revealed trends consistent with those observed at longer exposure times for both Chl *a* and carotenoids (Figure 5A–D). Regarding Dtx, the highest concentrations were again found in the double KOs, followed, in decreasing order, by *zep3*, *cpsrp54*, and WT (Figure 6A). However, the difference in Dtx concentration between *zep3* single mutants and double KO mutants was small and not statistically significant at the 0.5 h time point. This is in contrast with the differences in NPQ development observed between the *zep3* and double KO mutants during 10 min of moderate blue light intensities (Figure 4B,C). Because of the reported linear relationship between Dtx concentration and NPQ levels, a difference in Dtx concentration was also expected at the 0.5 h time point [50,51,52]. However, NPQ was not measured in the cultures that were sampled for pigment analysis, and it is unknown whether similar levels of Dtx provide similar photoprotective capabilities in the *zep3* and double KOs after ML treatment for 0.5 h. Schumann et al. [57] and Lepetit et al. [58] both reported that a linear relationship between NPQ and Dtx was true only until a certain Dtx concentration was reached (the threshold varied between light treatments). This observation can be explained by the fact that only protein-bound Dtx present in the Fx Chl *a*/*c*-binding protein (FCP) complexes takes part in NPQ, and that a major part of newly formed xanthophyll cycle pigments as a response to sustained light stress is located in a lipid shield around the FCPs [59]. Therefore, not only the amount of Dtx, but also the localization of the pigment will affect the photoprotective capabilities of the cells when exposed to sustained light stress [59]. Differences between *zep3* and double KO mutants became evident after 2 h of ML exposure, at which point the double KO mutants had accumulated 26% more Dtx than the *zep3* single mutants (Figure 6A). However, the concentration was still approximately 40% lower than in double KO mutants treated with ML for 6 and 10 h (Figure 5A). These results indicate that prolonged exposure of the double KO to increased light intensities is necessary when using ML (Figure 5A and Figure 6A) instead of HL [30] for accumulation of high levels of Dtx. 

### 2.6. Stability of Dtx in LL Conditions

Dtx accumulated during light stress conditions has previously been reported to be completely stable when *P. tricornutum zep3* mutants have been returned to lower light intensities for 0.5 h at 70 photons m^−2^ s^−1^ [32] and for up to 2 h at 35 photons m^−2^ s^−1^ [30], respectively. To assess whether the stability of accumulated Dtx observed in *zep3* mutants also applied to the double KOs, and to enable direct comparison with *zep3*, *cpsrp54,* and WT, the cultures were transferred back to LL after the 2 h ML treatment and sampled for pigment analysis (Figure 6). The complete back conversion of Dtx to Ddx in WT and *cpsrp54* cultures took place as expected after being returned to LL for 2 h, but surprisingly, a significant loss of Dtx was also observed for *zep3* single mutants and the double KO mutants after 2 h in LL (Figure 6A,B). Returning the *zep3* and double KO cultures to LL for 2 h caused an average loss of 39% and 32%, respectively, compared with the Dtx concentration after 2 h in ML (Figure 6A). A similar decrease in Dtx concentration was reported by Græsholt et al. in *zep3* cultures 6 h after being returned to LL. The loss was suggested to be caused by a 40% increase in cell number, causing Dtx to be distributed between daughter cells and thereby lowering the amount of Dtx per cell [30]. In the current study, there was only an approximate 15% increase in cell number (Supplementary Table S2) between the two harvesting time points for both *zep3* and double KO mutants, meaning that the decrease in Dtx concentration on a per cell basis can only be partly explained by an increase in cell number. Dtx concentration expressed per culture volume rather than per cell confirmed that Dtx was lost from the culture rather than just redistributed between daughter cells (Supplementary Figure S5). The Dtx concentration in µg/L indicated a 30% loss of Dtx in *zep3* cultures and a 22% loss of Dtx in the double KO cultures. The use of light sources with different light spectra (cool daylight [30] versus neutral light (this study)) and light meters equipped with different sensors (cosine-corrected/planar sensor [30] versus spherical sensor (this study)) to provide a LL intensity measured to 35 µmol photons m^−2^ s^−1^ are likely the main reasons for the observed differences in Dtx stability between the study by Græsholt et al. [30] and this study. A comparison of the light meters with the two different sensors revealed that the light intensity measured with the spherical sensor was twice as high as the intensity measured with the cosine-corrected sensor. Because of the above-described differences in light quality and light sensors, a higher number of photons were available for absorption by the photosynthetic apparatus of *P. tricornutum* in the previous study compared with this study [60]. Ware and coworkers [32] also used higher light intensities (70–75 µmol photons m^−2^ s^−1^) compared with this study as the low intensity light condition when analysing the presence of Dtx in *zep3* mutants before and after exposure to high-intensity light. Both these previous studies with *zep3* mutants detected substantial Dtx production in low-intensity light, also before exposing them to high-intensity light [30,32]. Their results indicate that Ddx will continue to be converted to Dtx even after being removed from the high-intensity light if the light intensity is not sufficiently lowered. The light quality and intensity (35 µmol photons m^−2^ s^−1^) used in this study and in a recent study by Giossi and coworkers (30 µmol photons m^−2^ s^−1^) [49] did not trigger Dtx production, implying that the conversion from Ddx to Dtx ceased when transferred back to these levels of low intensity light.

In addition to cell division, an explanation for the decrease in Dtx in *zep3* and double KO cultures after being returned to LL might be that ZEP2 can also perform the epoxidation reaction, converting Dtx to Ddx, although with low efficiency. Both *P. tricornutum* ZEP2 and ZEP3 have previously been shown to be able to restore ZEP activity and a functional xanthophyll cycle in the *Arabidopsis thaliana zep* mutant *npq2*, but ZEP2 had a broader substrate specificity than ZEP3 [61]. Consistent with this possibility, Ddx concentrations increased in both *zep3* and double KO mutants after 2 h in LL following ML exposure (Figure 6B). In addition, the DES index decreased, whereas the total pool of Ddx + Dtx was highly similar at the two latter time points for *zep3* and the double KOs (Figure 6E,F). However, the pigment analysis cannot discriminate between de novo synthesised Ddx and Ddx that has been converted back from Dtx. Alternatively, or additionally, carotenoid oxygenases, key players in carotenoid cleavage [62], might be involved in the degradation of Dtx in LL. However, the carotenoid oxygenases in *P. tricornutum* and other diatoms have not been functionally characterized. 

### 2.7. Isolation of Non-Transgenic zep3cpsrp54 KO Lines from the Four Independent Mutant Lines

**Figure 7:**
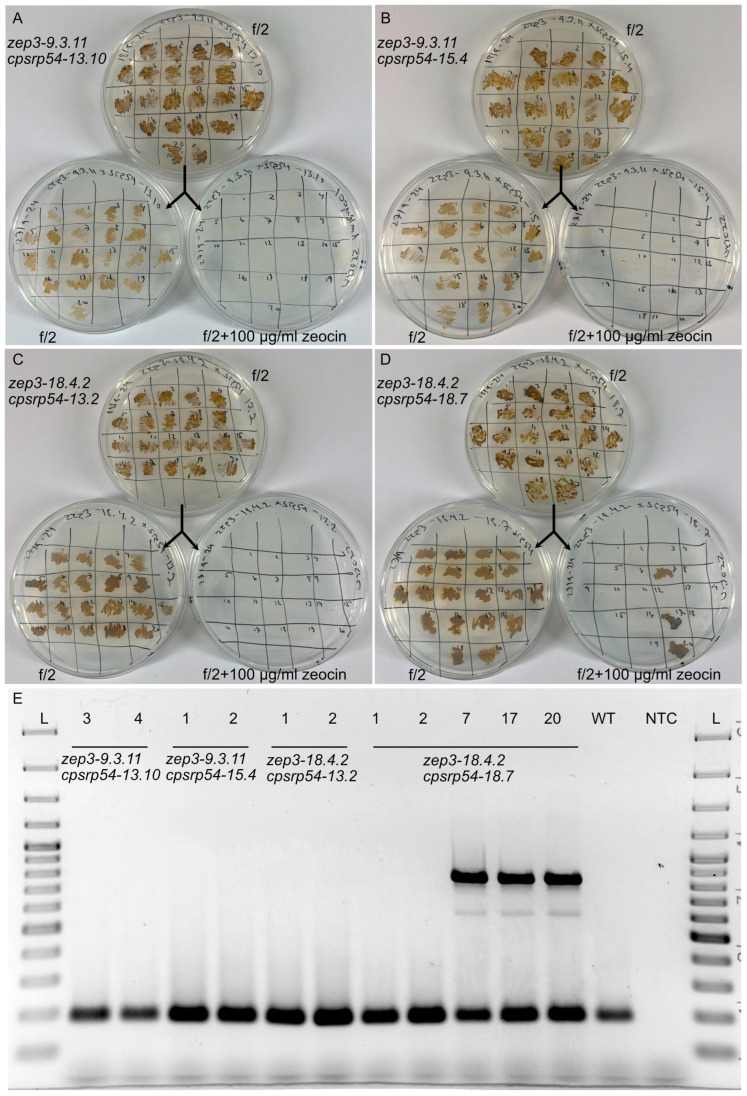
Identification of non-transgenic *zep3cpsrp54* double KO mutants. Twenty colonies derived from single cells from (**A**) *zep3-9.3.11cpsrp54-13.10*, (**B**) *zep3-9.3.11cpsrp54-15.4*, (**C**) *zep3-18.4.2cpsrp54-13.2*, and (**D**) *zep3-18.4.2cpsrp54-18.7* cultures were patched on 50% seawater (SW) f/2 agar plates. After around two weeks of growth, material was transferred to new 50% SW f/2 agar plates without zeocin or with zeocin (100 µg/mL). Only colonies still containing the pPtPuc3m-Cas9_sgRNA plasmid can survive on selection plates containing 100 µg/mL zeocin since the plasmid contains the *ShBle* gene conferring resistance to zeocin [44]. The presented pictures were taken eleven days after patching. (**E**) Loss of pPtPuc3m-Cas9_sgRNA plasmid was verified by the inability to amplify a PCR product from the plasmid’s origin of replication (855 bp). A genomic control fragment (Phatr2_52110; 188 bp) was co-amplified as a positive control. Two colonies from each double KO line no longer able to grow on selection plates were selected for PCR verification. In addition, three colonies (colony 7, 17, and 20) from line *zep3-18.4.2cpsrp54-18.7* that could still grow on selection plates were included as positive controls, and WT was included as a negative control. Numbers above lanes correspond to colony numbers on the agar plates. Abbreviations used are L: Ladder (GeneRuler 1 kb Plus DNA Ladder (ThermoFisher Scientific)); WT: Wild-type *P. tricornutum*; NTC: No template control.

Non-transgenic mutants created using the CRISPR/Cas9 technology present regulatory advantages since gene-edited organisms without foreign DNA are excluded from GMO regulations in a range of different countries outside of the EU, such as the UK, USA, Brazil, Argentina, Australia, Israel, India, and Japan [63], making commercialization of products from such mutants more accessible [64,65]. Several reports also indicate an increased consumer acceptance of products from non-transgenic gene-edited organisms compared with transgenic GMOs [66,67,68,69]. In this context, and because the phenotype of the *zep3cpsrp54* double KOs implies that these mutants might be of interest as a commercial production line of Dtx, non-transgenic single cells from each of the four *zep3cpsrp54* double KO lines were isolated as described previously [44] around 1 year after the double KOs had been created. Antibiotics had not been included in the growth medium during this time, and previous work has shown that diatom plasmids are lost in the majority of the cells when cultivated in the absence of selection pressure [42,44,70]. Twenty single cells from each of the four double KO mutant lines were included in the screening process, and of these, only three single cells from the *zep3-18.4.2cpsrp54-18.7* line were still able to grow on selective media (Figure 7A–D), indicating that these cells had retained the pPtPuc3m-Cas9_sgRNA plasmid containing the *SheBle* gene, enabling growth on zeocin-containing medium [71]. The loss or presence of the pPtPuc3m-Cas9_sgRNA plasmid was further confirmed in selected cells by attempting to amplify a PCR product (855 bp) from the plasmid’s origin of replication (Figure 7E). Co-amplification of a genomic fragment (188 bp) was performed as a positive control. Confirmed non-transgenic *zep3cpsrp54* double KOs are planned to be used in future research projects to investigate their potential as a large-scale Dtx production line. 

## 3. Materials and Methods

### 3.1. P. tricornutum Wild-Type and zep3, cpsrp54 and zep3cpsrp54 Mutant Lines

The *P. tricornutum* wild-type (WT) strain (derived from the genome sequenced clone Pt1 8.6 [41]) was obtained from the culture collection of the Provasoli-Guillard National Center for Culture of Marine Phytoplankton, Bigelow Laboratory for Ocean Sciences, USA. The *cpsrp54-11*, *cpsrp54-20, zep3-9.3* (*zep3-1* in [30]), and *zep3-18.4* (*zep3-2* in [30]) KO mutants were published previously and had been created with the aid of the CRISPR/Cas9 technology using a *P. tricornutum* culture derived from the WT strain described above [30,46]. *CpSRP54* and *ZEP3* have Draft IDs Phatr2_35185 and Phatr2_10970, respectively. 

The *zep3cpsrp54* double KO mutants were created by CRISPR/Cas9-mediated mutagenesis using the two previously published *zep3* lines as a starting point [30]. First, non-transgenic zep3 cells (cells that had lost the pPtPuc3m diaCas9_sgRNA plasmid used for CRISPR/Cas9 gene editing of the *ZEP3* gene) were isolated as described by Sharma and coworkers [44]. Cells from two *zep3* colonies (colony number 11 derived from a single cell from the *zep3-9.3* culture and colony 2 derived from a single cell from the *zep3-18.4* culture (Supplementary Figure S1)) no longer able to grow on 50% seawater (SW) F/2 agar plates with 100 µg/ml zeocin were selected and used to start up new cultures (*zep3-9.3.11* and *zep3-18.4.2*). The inability to grow on zeocin-containing agar plates indicated loss of the pPtPuc3m diaCas9_sgRNA plasmid containing the *ShBle* gene conferring resistance to zeocin. The *zep3-9.3.11* and *zep3-18.4.2* lines were subjected to Nanopore genome sequencing (see Section 3.2) to confirm that the two *zep3* lines contained bi-allelic mutations and that the pPtPuc3m diaCas9_sgRNA plasmid was no longer present in these cell cultures. The pPtPuc3m diaCas9_sgRNA plasmid expressing the Cas9 protein and a single guide RNA (sgRNA) targeting the *CpSRP54* gene was delivered to the *zep3* lines by bacterial conjugation as described previously [44]. The selected target site was the same as used previously to create *cpsrp54* single mutants [43,46]. Cloning of the CpSRP54-specific adapter into the sgRNA of the pPtPuc3m diaCas9_sgRNA vector and screening, identification, and isolation of cells containing bi-allelic mutations in the *CpSRP54* gene in the *zep3* background were also performed according to published methods [44,71]. Four *zep3cpsrp54* double KO mutants with small indels in both alleles were selected for phenotypic characterization. Oligonucleotides used for the creation of the CpSRP54-specific adapter inserted into the sgRNA of the pPtPuc3m diaCas9_sgRNA vector and primers used for screening purposes are listed in Nymark et al. [43]. After characterization of the *zep3cpsrp54* double KO mutants, non-transgenic lines were isolated as described before [44]. Loss of the pPtPuc3m diaCas9_sgRNA plasmid was verified by PCR on lysates from cells no longer capable of growing on zeocin-containing agar plates. The PCR reactions included primer pairs able to cause amplification of a PCR fragment from the plasmid’s Ori (origin of replication) and a genomic fragment from the Phatr2_52110 gene (positive control) in the same reaction tube. Lysates from WT (negative control) and *zep3cpsrp54* cells, still able to grow on selective media (positive controls), were also used as templates for the PCR reactions. The primer sequences are published in Sharma et al. [44]. 

### 3.2. Nanopore Sequencing of zep3 Lines

DNA was extracted from *zep3-9.3.11* and *zep3-18.4.2* cultures as previously described [72]. DNA libraries for Nanopore sequencing were prepared from 1 µg DNA using the Native Barcoding Kit 24 V14 (Oxford Nanopore Technologies SQK-NBD114.24, Oxford, UK) according to the manufacturer’s instructions in the Ligation sequencing gDNA – Native Barcoding Kit 24 V14 protocol (Version: NBE_9169_v114_revQ_15Sep2022-minion). In short, the DNA was repaired and end-prepped, and native barcodes were ligated. The barcoded samples were pooled, and sequencing adaptors were ligated. Libraries were loaded onto a MinION R10.4.1 Flow Cell and sequenced using a MinION MK1B device. Data acquisition and real-time base calling were carried out by the MinKNOW for MinION Mk1B software (version 24.02.8). A Canu assembler was used to create contigs that covered the *ZEP3* genomic region and to identify the indels [73]. Only MinION sequencing reads larger than 30,000 bp were used in the analysis. 

### 3.3. Light Conditions

*P. tricornutum* WT and mutant lines were grown at 15 °C, unless stated otherwise, in F/2 medium [74] made with 0.2 μm sterile filtered and autoclaved seawater from the Trondheim fjord. Microalgae cultivation was performed in 75 cm^2^ sterile Falcon polystyrene flasks (Corning Incorporated—Life Sciences, Durham, NC, USA) in a growth chamber equipped with neutral white LEDs (4000 K). The experimental light conditions used were 35–40 μmol photons m^−2^ s^−1^ (low light (LL)), 200–230 μmol photons m^−2^ s^−1^ (medium light (ML)), or 450–500 μmol photons m^−2^ s^−1^ (high light (HL) of continuous light, unless otherwise stated. The light intensity was measured with a ULM-500 (Walz, Effeltrich, Germany) light meter equipped with a spherical sensor.

### 3.4. Growth Rates

LED-based miniature photobioreactors (Nanocosm, Norwegian Creations, Trondheim, Norway [75]) were programmed to provide white light of 35, 70, 100, 150, 200, and 450 μmol photons m^−2^ s^−1^ for the cultivation of WT and *zep3cpsrp54* mutant lines. The cells were grown in 24-well plates (2 mL of culture in each well) at a starting concentration of 30,000 cells mL^−1^ at 10 °C and 15 °C. Growth was measured indirectly by recording the daily increase in in vivo Chl *a* fluorescence (IVF; Ex: 460 nm, Em: 680 nm) for nine days. IVF was measured using a Tecan Spark plate reader at five different points in each well. The averaged IVF values from four biological replicates from each line were used to plot growth curves, and the cell division rates were calculated from the exponential part of the curves.

### 3.5. Measurements of Photosynthetic Parameters

The photosynthetic parameters, F_v_/F_m_ or photosynthetic (photosystem II (PSII)) efficiency (calculated as (F_m_ − F_0_)/F_m_), the maximum relative electron transport rate (rETR_max_; photosynthetic capacity), the maximum light utilisation coefficient (alpha), and the light saturation index (Ek = rETR_max_/alpha) were calculated based on measurements of variable in vivo Chl *a* fluorescence using a Multi Color-PAM fluorometer (Walz, Effeltrich, Germany) as described in Græsholt et al. [30]. The measurements were performed in WT and *zep3cpsrp54* lines after acclimation to LL, ML, and HL for 1 week. The samples were dark incubated for 5 min before the first measurement. Rapid light curves (RLCs) were obtained by exposing the samples to 15 stepwise increasing irradiances of 0–1315 μmol photons m^−2^ s^−1^ (blue light (440 nm)). For the LL-acclimated WT and *zep3cpsrp54* lines cultures, NPQ was calculated as (i) a function of the stepwise increasing light intensity using the F_m_ and F_m′_ values generated during measurements of the RLCs, and (ii) as a function of time where the cells were exposed to 6 min of high-intensity blue light (470 μmol photons m^−2^ s^−1^), immediately followed by a 6 min recovery period in low-intensity blue light (8 μmol photons m^−2^ s^−1^). In addition, NPQ induction was estimated as a function of time (10 min) at a blue light intensity of 58, 124, 245, and 470 μmol photons m^−2^ s^−1^ in LL-acclimated WT, *cpsrp54*, *zep3*, and *zep3cpsrp54* lines. NPQ was calculated as (F_m_/F_m′_) – 1. Three biological replicates were included for all experiments.

### 3.6. Pigment Analysis

Pigment analysis, including quantification of diatoxanthin, were performed on samples from two different light experiments. In the first experiment, LL-acclimated WT, *cpsrp54*, *zep3*, and *zep3cpsrp54* lines were exposed to 6 and 10 h of ML. In the second experiment, LL-acclimated cultures were treated with ML for 0.5 and 2 h before being returned to LL (rLL) for 2 h. Three biological replicates were included for each line in both experiments. For each biological replicate, samples for pigment analysis were taken successively from the same culture flask. Samples were harvested by filtration onto 25 mm diameter Whatman GF/F filters and stored at −80 °C prior to pigment extraction and analysis. Pigments were extracted in dark glass vials from frozen filters containing 40–60 million microalgal cells, using 1.5 mL of cold methanol. After flushing the vials with nitrogen and sealing them with Teflon-lined screw caps, they were incubated at −20 °C for 24 h. Following gentle mixing, the extracts were filtered through PTFE syringe filters (13 mm Ø, 0.22 µm pore size) and subjected to HPLC analysis using an HP Agilent 1100 Series HPLC system with DAD detector (Agilent, Santa Clara, CA, USA) as described previously [76,77]. Pigment concentrations, including diatoxanthin, were calculated on a per-cell basis. Cell concentrations were determined using a Multisizer 4e Coulter Counter (Beckmann Coulter, Indianapolis, IN, USA).

### 3.7. Statistics

A two-way ANOVA with Dunnett’s multiple comparison tests was carried out using GraphPad Prism software (version 10.4.1, GraphPad, Boston, MA, USA) to determine if there were significant differences (*p* < 0.05) for growth rates, pigment concentrations, and photosynthetic parameters measured in WT and mutant lines. 

## 4. Conclusions

The *zep3cpsrp54* double KO mutants display several features that suggest that these lines could be suitable as industrial production lines for Dtx in indoor photobioreactors with artificial lighting. Their growth rates were equal to WT cultures when cultivated in non-stressful light conditions (≤150 µmol photons m^−2^ s^−1^). This feature allows for rapid accumulation of biomass before inducing Dtx production by an increase in light intensity. Under ML intensity, the double KOs reached Dtx concentrations that were almost 1.5 times higher than in *zep3* mutants, and more than 7 times higher than in WT. The possibility of using more moderate light intensities to induce Dtx production is likely to allow better scalability and higher biomass densities in photobioreactors where uniform high-intensity light exposure is difficult to achieve [78]. In addition, Dtx induction using moderate light intensities instead of high light intensities can reduce culture heating and energy use, resulting in a healthier culture and cost savings [78,79,80]. The double KO cultures also displayed limited loss (22%) of Dtx when transferred from ML to LL. This feature ensures high yields of Dtx even after a time-consuming, large-scale harvesting process in low light or darkness has been carried out [31]. Combining the use of *zep3cpsrp54* double KOs for Dtx production with harvesting at low temperature may further minimize potential loss of Dtx by inhibiting enzymatic processes such as ZEP2 epoxidation or carotenoid oxygenase breakdown of carotenoids [49,81]. Finally, the double KOs are non-transgenic. This feature enables bypassing of GMO regulation in a wide range of countries outside of the EU and can reduce consumer scepticism, time, and cost before market entry [63,64,66,67,68,69]. While these results highlight several advantages of using the double KOs as production lines for Dtx, limitations remain. The light sensitivity of the double KOs indicates that these mutants are less suitable for outdoor production of Dtx due to an uncontrollable light environment. In addition, the double KOs have not yet been tested as Dtx producers at high cell densities in large-scale photobioreactors. To fully realize the production potential of the double KO mutants, future studies should include large-scale cultivation trials where cultivation conditions, harvesting techniques, and preservation methods are optimized. 

## Data Availability

The *ZEP3* and *CpSRP54* genes have Draft IDs Phatr2_10970 and Phatr2_35185, respectively. *Zep3*, *cpsrp54,* and *zep3cpsrp54* mutant strains can be shared for research purposes. Raw data generated in the present study, used for the calculation of pigment concentrations, DNA sequence analysis, photophysiological parameters, and cell division rates, are available on request.

## Supplementary Materials

The following supporting information can be downloaded at: https://www.mdpi.com/article/10.3390/md23110419/s1, Figure S1: Identification of non-transgenic *zep3* KO mutants; Figure S2: NPQ induction in *cpsrp54*, *zep3*, *zep3cpsrp54* and WT as a response to constant light of different light intensities; Figure S3: Pigment levels and DES index in *cpsrp54*, *zep3*, *zep3cpsrp54* and WT after a shift from LL to ML conditions; Figure S4: Pigment levels and DES index in LL-acclimated *cpsrp54*, *zep3*, *zep3cpsrp54* and WT cells after a shift to ML followed by being returned to LL; Figure S5: Dtx concentration presented as µg/L in LL-acclimated *cpsrp54*, *zep3*, *zep3cpsrp54* and WT cells after a shift to ML followed by being returned to LL; Table S1: Statistical analyses of differences in NPQ induction between *zep3cpsrp54* and the three other *P. tricornutum* lines (WT, *cpsrp54,* and *zep3*); Table S2: Cell concentrations at the different harvesting time points during the shorter-term ML exposure experiment.

## Abbreviations

The following abbreviations are used in this manuscript:

ZEP	Zeaxanthin epoxidase
CpSRP54	Chloroplast signal recognition particle 54
Chl	Chlorophyll
Fx	Fucoxanthin
Dtx	Diatoxanthin
Ddx	Diadinoxanthin
VDE	Violaxanthin de-epoxidase
LHC	Light-harvesting complex
KO	Knock out
NPQ	Non-photochemical quenching
PAM	Protospacer adjacent motifs
LL	Low light
ML	Medium light
HL	High light
WT	Wild-type
rETRmax	Maximum relative electron transport rate
Ek	Light saturation index
Alpha	Maximum light utilisation coefficient
DES	De-epoxidation state
PSII	Photosystem II
FCP	Fx Chl *a*/*c*-binding protein
IVF	In vivo Chl *a* fluorescence
RLC	Rapid light curve
