# Supplementary material for: Creating an Improved Diatoxanthin Production Line by Knocking Out CpSRP54 in the zep3 Background in the Marine Diatom Phaeodactylum tricornutum
Source: Mar Drugs. 2025 Oct 29;23(11):419. doi: 10.3390/md23110419 (PMC12654249; doi:10.3390/md23110419)
Supplement: Supplementary file 1 [file marinedrugs-23-00419-s001.zip › marinedrugs-3933165-supplementary.pdf]

Supplementary information

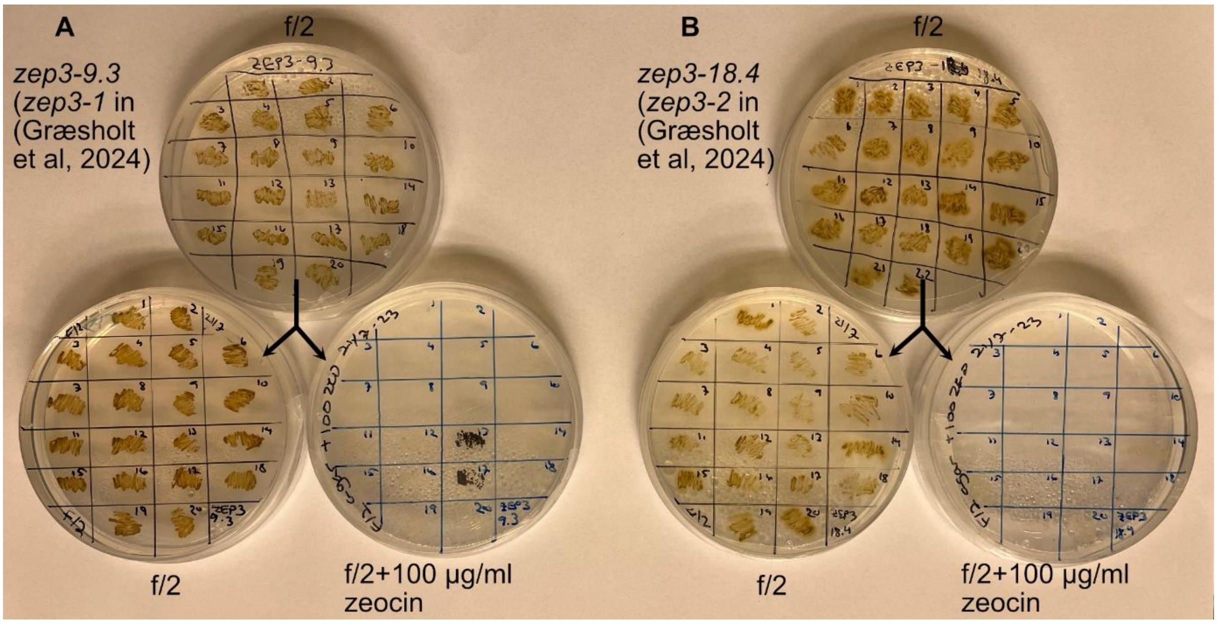

Figure S1. Identification of non-transgenic *zep3* KO mutants. 20 colonies derived from single cells from (A) *zep3-9.3* and (B) *zep3-18.4* cultures were patched on a 50% seawater (SW) f/2 agar plate. After around two weeks of growth, material was transferred to new 50% SW f/2 agar plates without zeocin (A and B, bottom left side) or with zeocin (A and B, bottom right side). Only colonies still containing the pPtPuc3m-Cas9_sgRNA plasmid can survive on selection plates containing 100 µg/ml zeocin. Presented pictures were taken twelve days after patching.

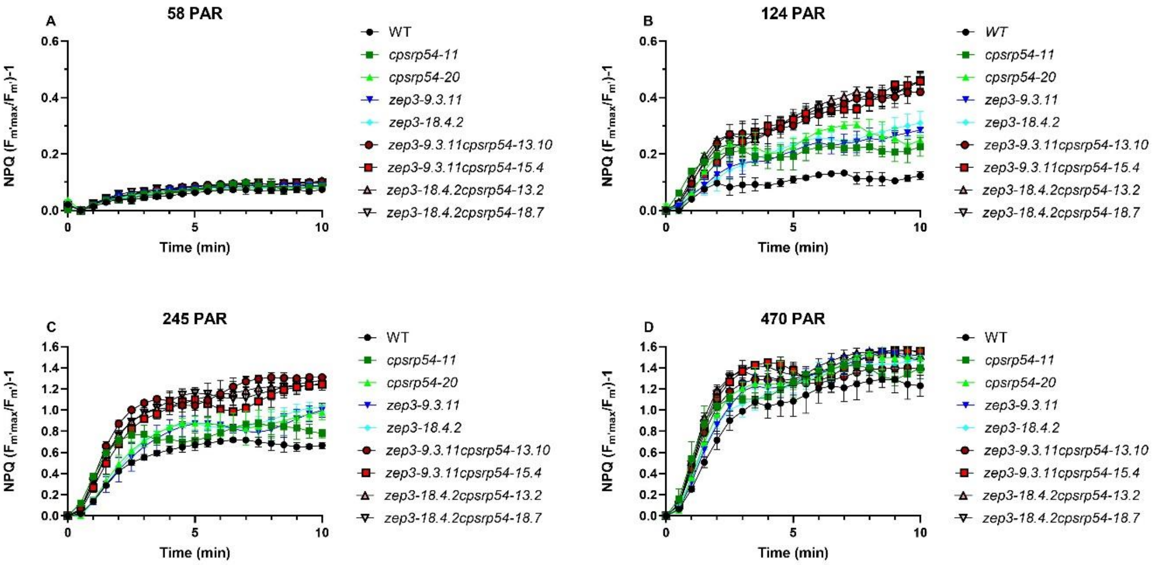

Figure S2. NPQ induction in *cpsrp54*, *zep3*, *zep3cpsrp54* and WT as a response to constant light of different light intensities. NPQ was calculated as a function of time where the cells were exposed to (A) 58 µmol photons m^{-2} s^{-1} blue light, (B) 124 µmol photons m^{-2} s^{-1} blue light, (C) 245 µmol photons m^{-2} s^{-1} and D) 470 µmol photons m^{-2} s^{-1} blue light for 10 minutes. Two different lines were included in the experiment for *cpsrp54* (*cpsrp54-11*, *cpsrp54-20*) and *zep3* (*zep3-9.3.11*, *zep3-18.4.2*), and four different lines were included for *zep3cpsrp54* (*zep3-9.3.11cpsrp54-13.10, zep3-9.3.11cpsrp54-15.4, zep3-18.4.2cpsrp54-13.2, zep3-18.4.2cpsrp54-18.7*). Three biological replicates were included for each of the mutant lines and WT. The graphs are based on the means of the replicates for each mutant or WT.

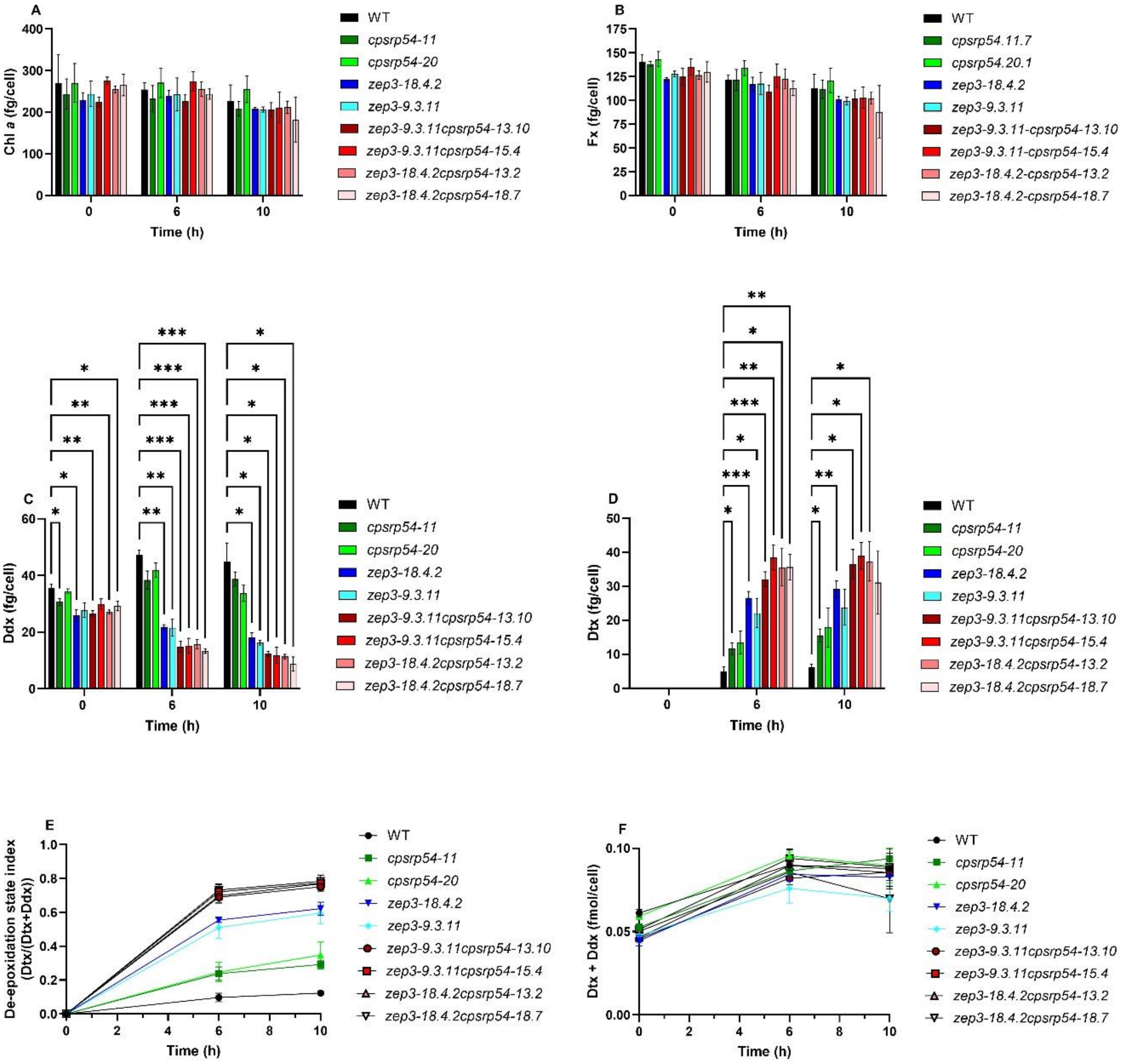

Figure S3. Pigment levels and DES index in *cpsrp54*, *zep3*, *zep3cpsrp54* and WT after a shift from LL to ML conditions. Cellular pigment content (fg/cell) are shown for (A) Chl *a*, (B) Fx, (C) Ddx, and (D) Dtx in *cpsrp54*, *zep3*, *zep3cpsrp54* and WT after exposure of LL (35 µmol photons m^{-2} sec^{-1}) acclimated cells (0 h) to 6 and 10 h of ML (200 µmol photons m^{-2} s^{-1}). Three biological replicates were included for WT and each of the mutant lines. Asterisks describe significant differences between mutants and WT as indicated by two-way ANOVA with Dunnett's multiple comparison tests ($P < 0.05$). (E) DES index (DES = Dtx/(Dtx + Ddx)) calculated from data shown in (C) and (D) after conversion to fmol/cell. (F) Changes in the total pool of Dtx+Ddx (fmol/cell) as a function of time after the shift from LL to ML.

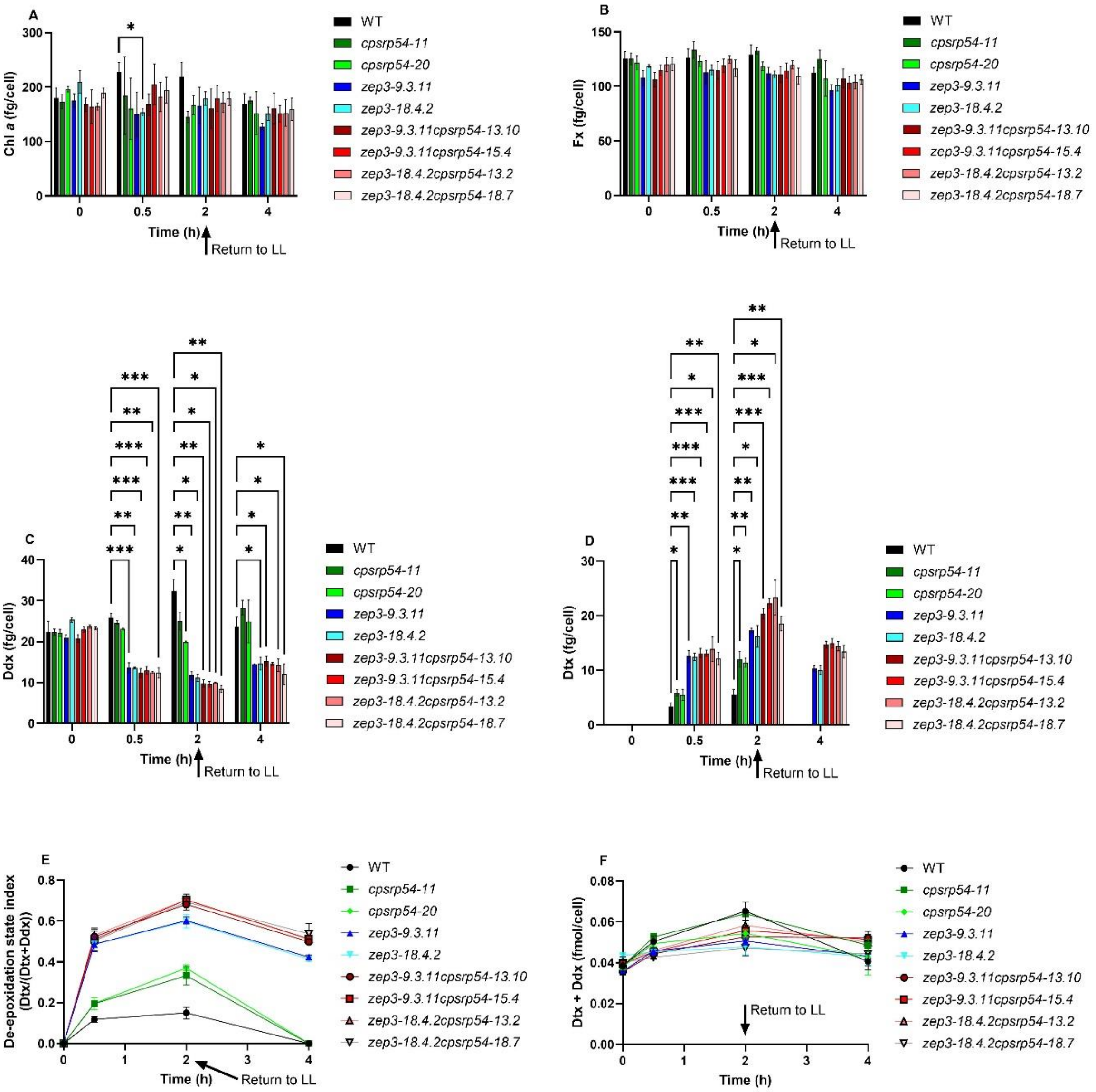

Figure S4. Pigment levels and DES index in LL acclimated *cpsrp54*, *zep3*, *zep3cpsrp54* and WT cells after a shift to ML followed by being returned to LL. Cellular pigment content (fg/cell) are shown for (A) Chl *a*, (B) Fx, (C) Ddx, and (D) Dtx in *cpsrp54*, *zep3*, *zep3cpsrp54* and WT after exposure of LL (35 µmol photons m^{-2} sec^{-1}) acclimated cells (0 h) to 0.5 and 2 h of ML (200 µmol photons m^{-2} s^{-1}). The cultures were returned to LL for 2 h after 2 h of ML treatment. Three biological replicates were included for WT and each of the mutant lines. Asterisks for figures A-D describe significant differences between mutants and WT as indicated by two-way ANOVA with Dunnett's multiple comparison tests ($P < 0.05$). (E) DES index (DES = Dtx/(Dtx + Ddx)) calculated from data shown in (C) and (D) after conversion to fmol/cell. (F) Changes in the total pool of Dtx+Ddx (fmol/cell) as a response to the different light treatments.

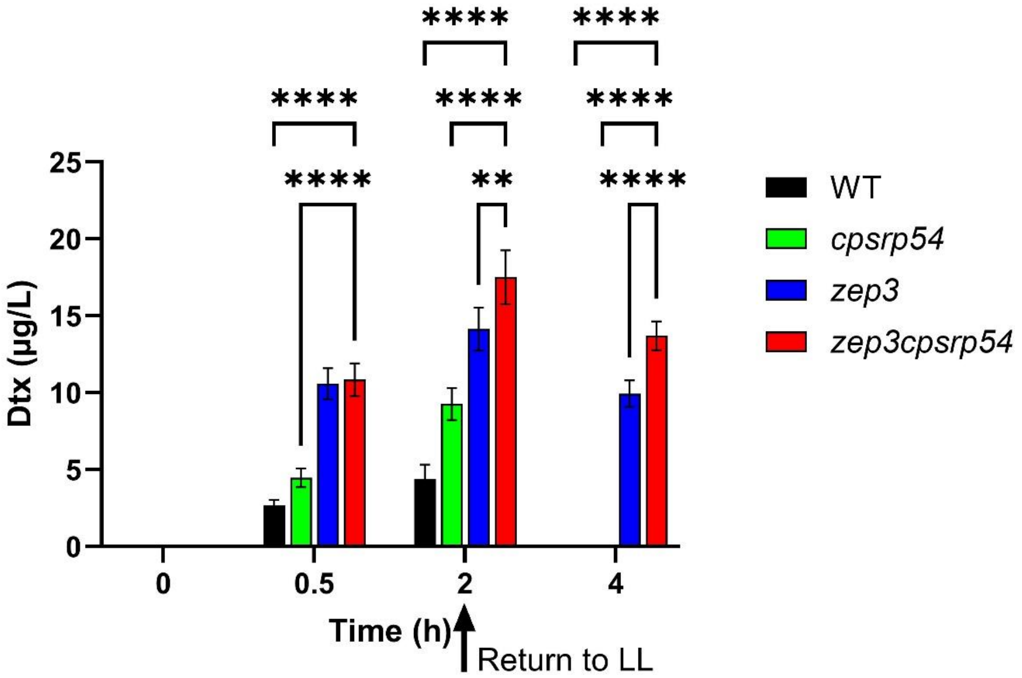

Figure S5. Dtx concentration presented as µg/L in LL acclimated *cpsrp54*, *zep3*, *zep3Cpsrp54* and WT cells after a shift to ML followed by being returned to LL. Dtx concentration is presented as µg/L for *cpsrp54*, *zep3*, *zep3cpsrp54* and WT after exposure of LL (35 µmol photons m^{-2} sec^{-1}) acclimated cells (0 h) to 0.5 and 2 h of ML (200 µmol photons m^{-2} s^{-1}). The cultures were returned to LL for 2 h after 2 h of ML treatment. Three biological replicates were included for each of the mutant lines. The graph is based on the means of all lines and replicates for each mutant or WT. Asterisks describe significant differences between *zep3cpsrp54* and the other lines as indicated by two-way ANOVA with Dunnett's multiple comparison tests ($P < 0.05$).

Table S1. Statistical analyses of differences in NPQ induction between *zep3cpsrp54* and three other *P. tricornutum* lines (WT, *cpsrp54* and *zep3*). NPQ was induced by 10 min of blue light exposure of 58, 124, 245 and 470 µmol photons m^{-2} s^{-1} for 10 minutes. Two different lines were included in the experiment for *cpsrp54* (*cpsrp54-11*, *cpsrp54-20*) and *zep3* (*zep3-9.3.11*, *zep3-18.4.2*), and four different lines were included for *zep3cpsrp54* (*zep3-9.3.11cpsrp54-13.10, zep3-9.3.11cpsrp54-15.4, zep3-18.4.2cpsrp54-13.2, zep3-18.4.2cpsrp54-18.7*) mutant lines. Three biological replicates were included for each of the mutant lines meaning that the total number of samples were n=6 for *zep3* and *cpsrp54*; n=12 for *zep3cpsrp54* and n=3 for WT. The results are based on all replicates for each mutant or WT. Significant differences between *zep3cpsrp54* and the other lines for each time point and light intensity were investigated by performing a two-way ANOVA with Dunnett's multiple comparison tests.

		**58 PAR**		**124 PAR**		**245 PAR**		**470 PAR**	
**Time (min)**	**Comparison**	**Summary**	**Adjusted P Value**	**Summary**	**Adjusted P Value**	**Summary**	**Adjusted P Value**	**Summary**	**Adjusted P Value**
**0**	*zep3cpsrp54* vs. WT	ns	0.9793						
**0**	*zep3cpsrp54* vs. *cpsrp54*	ns	>0.9999						
**0**	*zep3cpsrp54* vs. *zep3*	ns	0.4499						
**0.5**	*zep3cpsrp54* vs. WT			****	<0.0001	**	0.0032	*	0.0187

**0.5**	*zep3cpsrp54* vs. *cpsrp54*			ns	0.9344	ns	0.9738	ns	0.9996
**0.5**	*zep3cpsrp54* vs. *zep3*			ns	0.1449	****	<0.0001	ns	0.0508
**1**	*zep3cpsrp54* vs. WT	ns	0.1686	****	<0.0001	****	<0.0001	***	0.0002
**1**	*zep3cpsrp54* vs. *cpsrp54*	*	0.0442	ns	0.9993	ns	0.5196	ns	0.9635
**1**	*zep3cpsrp54* vs. *zep3*	ns	0.1457	**	0.0025	****	<0.0001	****	<0.0001
**1.5**	*zep3cpsrp54* vs. WT	ns	0.1094	****	<0.0001	****	<0.0001	***	0.0005
**1.5**	*zep3cpsrp54* vs. *cpsrp54*	ns	0.1141	ns	0.9022	ns	0.256	ns	0.5948
**1.5**	*zep3cpsrp54* vs. *zep3*	ns	0.5028	***	0.0004	****	<0.0001	****	<0.0001
**2**	*zep3cpsrp54* vs. WT	ns	0.1705	****	<0.0001	****	<0.0001	*	0.0111
**2**	*zep3cpsrp54* vs. *cpsrp54*	ns	0.3071	ns	0.3791	ns	0.0745	*	0.0145
**2**	*zep3cpsrp54* vs. *zep3*	ns	0.8499	***	0.0005	****	<0.0001	****	<0.0001
**2.5**	*zep3cpsrp54* vs. WT	ns	0.2634	**	0.0026	****	<0.0001	*	0.0239
**2.5**	*zep3cpsrp54* vs. *cpsrp54*	ns	0.6389	ns	0.3666	*	0.0103	***	0.0002
**2.5**	*zep3cpsrp54* vs. *zep3*	ns	0.6929	***	0.0004	****	<0.0001	***	0.0002
**3**	*zep3cpsrp54* vs. WT	ns	0.1836	**	0.0022	****	<0.0001	****	<0.0001
**3**	*zep3cpsrp54* vs. *cpsrp54*	ns	0.5592	ns	0.16	***	0.0005	**	0.0011
**3**	*zep3cpsrp54* vs. *zep3*	ns	0.7397	***	0.0004	***	0.0002	*	0.0106
**3.5**	*zep3cpsrp54* vs. WT	ns	0.0561	***	0.0007	****	<0.0001	**	0.0037
**3.5**	*zep3cpsrp54* vs. *cpsrp54*	ns	0.0782	ns	0.0591	****	<0.0001	*	0.0404
**3.5**	*zep3cpsrp54* vs. *zep3*	ns	0.3341	****	<0.0001	***	0.0002	*	0.017
**4**	*zep3cpsrp54* vs. WT	***	0.0007	****	<0.0001	****	<0.0001	*	0.0495
**4**	*zep3cpsrp54* vs. *cpsrp54*	*	0.0469	***	0.0002	***	0.0003	ns	0.0882
**4**	*zep3cpsrp54* vs. *zep3*	ns	0.7621	****	<0.0001	***	0.0003	***	0.0004
**4.5**	*zep3cpsrp54* vs. WT	**	0.0018	****	<0.0001	****	<0.0001	ns	0.0899
**4.5**	*zep3cpsrp54* vs. *cpsrp54*	*	0.0164	***	0.0004	**	0.0017	ns	0.3447
**4.5**	*zep3cpsrp54* vs. *zep3*	ns	0.7523	****	<0.0001	****	<0.0001	**	0.0069
**5**	*zep3cpsrp54* vs. WT	***	0.0002	****	<0.0001	****	<0.0001	ns	0.2078
**5**	*zep3cpsrp54* vs. *cpsrp54*	*	0.0212	**	0.0014	**	0.001	ns	0.5866
**5**	*zep3cpsrp54* vs. *zep3*	ns	0.9711	****	<0.0001	****	<0.0001	ns	0.2862
**5.5**	*zep3cpsrp54* vs. WT	****	<0.0001	****	<0.0001	****	<0.0001	ns	0.4599
**5.5**	*zep3cpsrp54* vs. *cpsrp54*	ns	0.0546	***	0.0006	***	0.0005	ns	>0.9999

**5.5**	*zep3cpsrp54* vs. *zep3*	ns	0.7084	****	<0.0001	****	<0.0001	ns	>0.9999
**6**	*zep3cpsrp54* vs. WT	***	0.0002	****	<0.0001	****	<0.0001	ns	0.5018
**6**	*zep3cpsrp54* vs. *cpsrp54*	ns	0.2031	***	0.0006	**	0.0013	ns	0.9967
**6**	*zep3cpsrp54* vs. *zep3*	ns	0.1671	****	<0.0001	****	<0.0001	ns	0.9583
**6.5**	*zep3cpsrp54* vs. WT	ns	0.0891	****	<0.0001	****	<0.0001	*	0.0486
**6.5**	*zep3cpsrp54* vs. *cpsrp54*	ns	0.2594	***	0.001	**	0.0015	ns	0.9891
**6.5**	*zep3cpsrp54* vs. *zep3*	*	0.0163	****	<0.0001	****	<0.0001	ns	0.993
**7**	*zep3cpsrp54* vs. WT	ns	0.2759	****	<0.0001	****	<0.0001	ns	0.1935
**7**	*zep3cpsrp54* vs. *cpsrp54*	ns	0.4918	**	0.0029	**	0.0032	ns	0.983
**7**	*zep3cpsrp54* vs. *zep3*	*	0.0431	****	<0.0001	****	<0.0001	ns	0.9997
**7.5**	*zep3cpsrp54* vs. WT	ns	0.0685	****	<0.0001	****	<0.0001	ns	0.2348
**7.5**	*zep3cpsrp54* vs. *cpsrp54*	ns	0.5331	**	0.0015	**	0.0016	ns	0.9993
**7.5**	*zep3cpsrp54* vs. *zep3*	ns	0.1279	****	<0.0001	****	<0.0001	ns	0.9748
**8**	*zep3cpsrp54* vs. WT	ns	0.2239	***	0.0005	***	0.0003	ns	0.0988
**8**	*zep3cpsrp54* vs. *cpsrp54*	ns	0.9495	***	0.0003	**	0.0014	ns	0.9258
**8**	*zep3cpsrp54* vs. *zep3*	ns	0.5101	****	<0.0001	****	<0.0001	ns	0.8889
**8.5**	*zep3cpsrp54* vs. WT	ns	0.1954	***	0.0002	**	0.0018	*	0.0342
**8.5**	*zep3cpsrp54* vs. *cpsrp54*	**	0.0035	***	0.0002	**	0.001	ns	0.2902
**8.5**	*zep3cpsrp54* vs. *zep3*	ns	0.1878	****	<0.0001	***	0.0002	ns	0.9796
**9**	*zep3cpsrp54* vs. WT	ns	0.1044	****	<0.0001	****	<0.0001	ns	0.1597
**9**	*zep3cpsrp54* vs. *cpsrp54*	***	0.0002	****	<0.0001	**	0.0019	ns	0.3142
**9**	*zep3cpsrp54* vs. *zep3*	ns	0.1426	****	<0.0001	***	0.0002	ns	0.8156
**9.5**	*zep3cpsrp54* vs. WT	*	0.0227	****	<0.0001	****	<0.0001	ns	0.1488
**9.5**	*zep3cpsrp54* vs. *cpsrp54*	****	<0.0001	****	<0.0001	**	0.0026	ns	0.3931
**9.5**	*zep3cpsrp54* vs. *zep3*	ns	0.0819	****	<0.0001	****	<0.0001	ns	0.4113
**10**	*zep3cpsrp54* vs. WT	***	0.0005	****	<0.0001	****	<0.0001	ns	0.0666
**10**	*zep3cpsrp54* vs. *cpsrp54*	***	0.0002	****	<0.0001	**	0.0039	ns	0.6246
**10**	*zep3cpsrp54* vs. *zep3*	*	0.0195	****	<0.0001	****	<0.0001	ns	0.7167

Table S2. Cell concentrations at the different harvesting time points during the shorter-term ML exposure experiment. LL acclimated *zep3 and cpsrp54* single KO mutants, *zep3cpsrp54* double KO lines and WT cultures were exposed to ML for 0.5 h and 2 h before being returned to LL for 2 h (2h rLL). Cell concentrations were calculated using a Multisizer 4e Coulter Counter (Beckmann Coulter, Indianapolis, IN, USA) and are presented as cells/ml. The cell numbers were used for the calculations of pigment concentrations per cell. The same cell numbers were used for the 0.5 h and 2h ML-treated cultures. Before transfer of LL-acclimated cultures to ML conditions some of the culture volume was transferred to new flasks, kept at LL and harvested at the end of the experiment.

	Cells mL^{-1}		
**Sample name**	**0.5h ML and 2h ML**	**2h rLL**	**LL**
**WT I**	727200	879200	923600
**WT II**	796800	899200	981600
**WT III**	829400	932800	1008800
***cpsrp54-11* I**	742800	886400	955200
***cpsrp54-11* II**	715600	778000	935200
***cpsrp54-11* III**	781200	931600	1113200
***cpsrp54-20* I**	823200	898400	1034400
***cpsrp54-20* II**	815200	927600	994000
***cpsrp54-20* III**	861200	938800	1099600
***zep3-9.3.11* I**	801600	924800	1053600
***zep3-9.3.11* II**	885200	1021200	1172800
***zep3-9.3.11* III**	841200	996000	1126000
***zep3-18.4.2* I**	822000	930800	1062800
***zep3-18.4.2* I**	847600	968000	1031200
***zep3-18.4.2* I**	857200	997600	1106400
***zep3-9.3.11cpsrp54-13.10* I**	850400	912400	1052000
***zep3-9.3.11cpsrp54-13.10* II**	887200	981200	1113200
***zep3-9.3.11cpsrp54-13.10* III**	824400	928400	1123600
***zep3-9.3.11cpsrp54-15.4* I**	812000	985200	1015600
***zep3-9.3.11cpsrp54-15.4* II**	850800	988800	1041200
***zep3-9.3.11cpsrp54-15.4* III**	828800	944400	1046400
***zep3-18.4.2cpsrp54-13.2* I**	819200	922200	979200
***zep3-18.4.2cpsrp54-13.2* II**	784800	978800	1030800
***zep3-18.4.2cpsrp54-13.2* III**	794400	998200	1070800
***zep3-18.4.2cpsrp54-18.7* I**	819200	931600	956800
***zep3-18.4.2cpsrp54-18.7* II**	831600	930400	996000
***zep3-18.4.2cpsrp54-18.7* III**	857600	918800	992000
